# Surface Characterization, Corrosion Resistance and in Vitro Biocompatibility of a New Ti-Hf-Mo-Sn Alloy

**DOI:** 10.3390/ma9100818

**Published:** 2016-10-04

**Authors:** Raluca Ion, Silviu Iulian Drob, Muhammad Farzik Ijaz, Cora Vasilescu, Petre Osiceanu, Doina-Margareta Gordin, Anisoara Cimpean, Thierry Gloriant

**Affiliations:** 1 Department of Biochemistry and Molecular Biology, University of Bucharest, 91-95 Spl. Independentei, Bucharest 050095, Romania; rciubar@yahoo.com; 2 Institute of Physical Chemistry “Ilie Murgulescu”, Romanian Academy, Bucharest 060021, Romania; sidrob.icf@gmail.com (S.I.D.); cora_vasilescu@yahoo.com (C.V.); petre.osiceanu@yahoo.com (P.O.); 3 Institut des Sciences Chimiques de Rennes, UMR CNRS 6226, INSA Rennes, 20 avenue des Buttes de Coësmes, Rennes 35708, France; farzik98@gmail.com (M.F.I.); doina.gordin@insa-rennes.fr (D.-M.G.); thierry.gloriant@insa-rennes.fr (T.G.)

**Keywords:** biomedical alloy, native passive film, electrochemical behavior, corrosion resistance, endothelial cell behavior

## Abstract

A new superelastic Ti-23Hf-3Mo-4Sn biomedical alloy displaying a particularly large recovery strain was synthesized and characterized in this study. Its native passive film is very thick (18 nm) and contains very protective TiO_2,_ Ti_2_O_3_, HfO_2_, MoO_2_, and SnO_2_ oxides (XPS analysis). This alloy revealed nobler electrochemical behavior, more favorable values of the corrosion parameters and open circuit potentials in simulated body fluid in comparison with commercially pure titanium (CP-Ti) and Ti-6Al-4V alloy taken as reference biomaterials in this study. This is due to the favorable influence of the alloying elements Hf, Sn, Mo, which enhance the protective properties of the native passive film on alloy surface. Impedance spectra showed a passive film with two layers, an inner, capacitive, barrier, dense layer and an outer, less insulating, porous layer that confer both high corrosion resistance and bioactivity to the alloy. In vitro tests were carried out in order to evaluate the response of Human Umbilical Vein Endothelial Cells (HUVECs) to Ti-23Hf-3Mo-4Sn alloy in terms of cell viability, cell proliferation, phenotypic marker expression and nitric oxide release. The results indicate a similar level of cytocompatibility with HUVEC cells cultured on Ti-23Hf-3Mo-4Sn substrate and those cultured on the conventional CP-Ti and Ti-6Al-4V metallic materials.

## 1. Introduction

Multifunctional β Ti-based alloys elaborated with biocompatible alloying elements have become an important field of investigation for biomedical applications due to their advantageous characteristics, such as low modulus and high elastic recovery [1,2,3,4]. Over the years, several new kinds of metastable β Ti-based alloys with superior superelastic properties have been introduced owing to distinct compositional modifications and/or targeted alloy design. For example, metastable β Ti-Nb and β Ti-Zr based alloys have been found to exhibit large superelastic recovery strain at room temperature through reversible stress-induced martensitic transformation between parent β phase (body centered cubic structure) and martensite α″ phase (orthorhombic structure) [2,5,6,7].

In a previous work [8], a new Ti-23Hf-3Mo-4Sn superelastic alloy was elaborated and characterized. With this alloy composition, outstanding combination of high strength (~1 GPa), low Young’s modulus (55 GPa) and large recovery strain of about 4% were achieved. These mechanical properties make this newly developed Ti-23Hf-3Mo-4Sn alloy very promising for the development of new biocompatible devices such as superelastic self-expanding stents. One important feature for biomedical devices is the corrosion resistance, which must necessarily be high in order to avoid the release of metallic products and to prevent inflammatory responses. The corrosion resistance of Ti is well-known; its thin native passive film ensures protection on a very large potential and pH range [9]. Hafnium [9] has a native passive film consisting of HfO_2_ oxide; this oxide is very resistant from pH ≈ 4 to pH ≈ 16 in a potential domain comprising −1.8 V and +1.8 V (vs. SHE); thus, it is an improvement of the protective capacity is expected by alloying Hf with Ti. Tin [9] is immune and then is passive for the whole pH range (from −2 till 16) and a large potential domain; its resistant SnO_2_ will reinforce the passive film on Ti surface by alloying. Molybdenum [9] has a large immunity potential range (from −2 V till −0.4 V vs. SHE) and will shift the corrosion potentials of its alloys with Ti to nobler direction. Thus, alloying Ti with Hf, Sn and Mo can lead to excellent corrosion resistance [10]. There is only little information about the titanium binary alloys with hafnium. Jeong et al. [11] applied nanotubes on binary Ti-xHf (x = 10%, 20%, 30% and 40%) alloy surfaces for dental use and the best behavior could be observed for the Ti-20Hf alloy. Ternary titanium alloy with Ta and Hf and Ti-35Ta-xHf (x = 3–15 wt %) were coated with nanotubular structures of TiN/ZrN to increase their biocompatibility [12]. A new quaternary equiatomic Hf-25Sc-25Ti-25Zr alloy [13] was processed by compression deformation and annealed and showed a high thermal stability.

The main aim of this study is to characterize, for the first time, a novel Ti-23Hf-3Mo-4Sn alloy in terms of its native passive film composition and thickness (by X-ray photoelectron spectroscopy—XPS); the electrochemical stability and corrosion resistance (by cyclic and linear polarization, electrochemical impedance spectroscopy—EIS) of the interface between the new alloy and simulated body fluid—SBF, its biocompatibility by in vitro tests with Human Umbilical Vein Endothelial Cells (HUVECs), in terms of cell viability and proliferation, phenotypic marker expression and nitric oxide release. The values of all the electrochemical and corrosion parameters prove a nobler electrochemical behavior and a higher protective capacity of the new superelastic Ti-23Hf-3Mo-4Sn alloy by comparison with commercially pure titanium (CP-Ti, grade 2) and Ti-6Al-4V (grade 5 ELI) alloy. CP-Ti and Ti-6Al-4V medical grades were chosen as control biomaterials in this study because they are regularly used for cardiovascular devices such as pacemakers, heart valves, catheters, vascular clips. Endothelial cells in contact with the three analyzed metallic samples display excellent growth capacity and characteristic phenotype and functions. Furthermore, this alloy was previously shown to possess an equiaxed β-phase grain microstructure and a perfect superelastic behavior with a particularly large recovery strain of about 4% [8].

## 2. Results and Discussion

### 2.1. Composition and Thickness of the Alloy Native Passive Film

**Figure 1:**
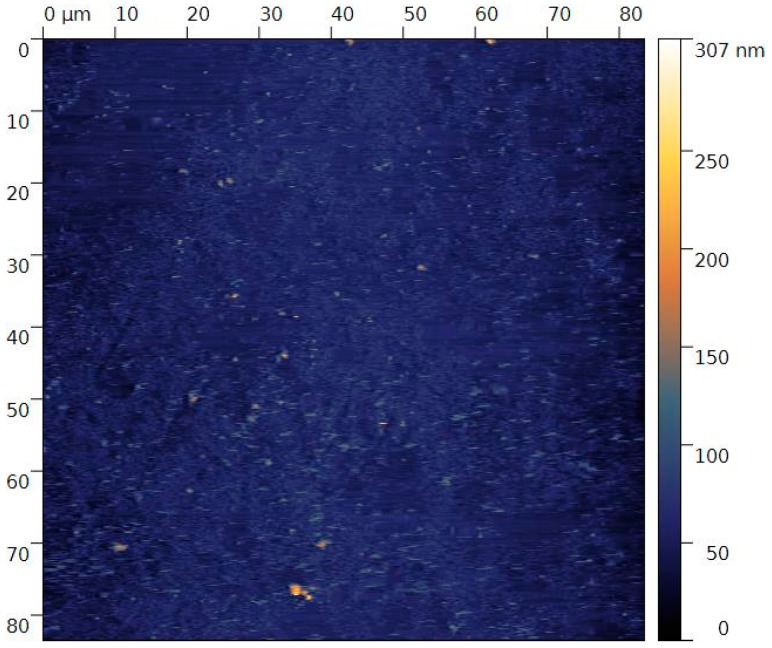
Example of AFM map (tapping mode) realized for the evaluation of the superficial roughness.

Before analysis, the roughness of the mirror polished samples was evaluated by atomic force microscopy (AFM). An example of AFM map is presented in Figure 1. From the different AFM maps realized, a uniform roughness was obtained and the roughness value, Ra, was measured to be 14 nm ± 3 nm.

**Figure 2:**
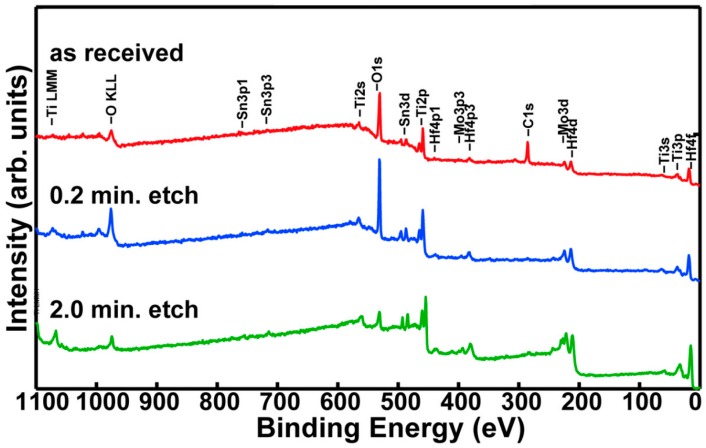
The XPS wide scan/surveys superimposed spectra for the as received Ti-23Hf-3Mo-4Sn alloy and after 0.2 and 2 min etching.

The XPS survey spectrum shown in Figure 2 reveals that the thickness of the native passive film is ~18.0 ± 1.0 nm [14]. Quantitative assessment indicates that the cation relative concentrations are Ti = 69.2%; Hf = 23.5%; Mo = 1.8%; Sn = 5.5%. It results that the surface is depleted of Mo and enriched in Sn (Mo diffusion from surface to the subsurface region is accompanied by segregation of Sn to the outermost surface layer).

**Figure 3:**
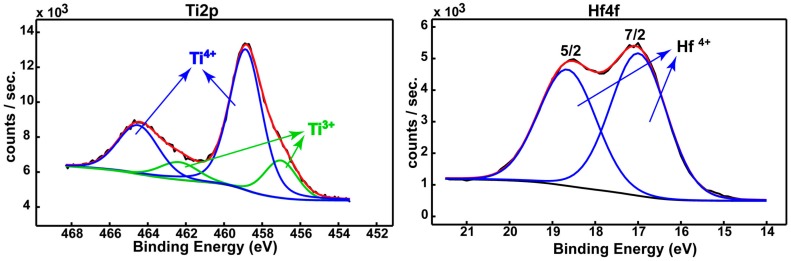

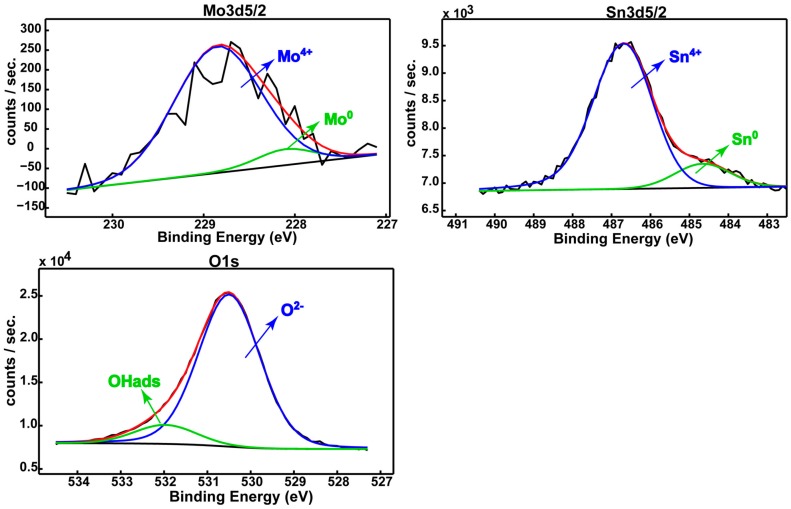
Deconvoluted spectra for the constitutive elements of the native passive film existing on the Ti-23Hf-3Mo-4Sn alloy surface.

High-resolution spectra (Figure 3) were collected in order to highlight the chemistry of the detected elements [15,16], while Table 1 and Table 2 summarize the associated quantitative assessments.

The deconvoluted spectra identified the constituent elements of the native passive film (Ti 2p, Hf 4f, Mo 3d, Sn 3d, and O 1s) after their binding energies (Table 1). Table 2 evidenced that: Ti occurs as a mixture of TiO_2_ (78.7%) and Ti_2_O_3_ (21.3%); Hf is fully oxidized (HfO_2_); Mo shows a mixture of MoO_2_ (91.6%) and metallic Mo (8.4%); Sn exhibits a mixture of SnO_2_ (87.0%) and metallic Sn (13.0%). The thicknesses of the individual oxides are quite different, HfO_2_ being the main contributor to the overall thickness of the film on the new alloy surface.

XPS results indicate that the native passive film on the new Ti-23Hf-3Mo-4Sn alloy surface is thicker, more compact, reinforced, and contains additional HfO_2_, MoO_2_, SnO_2_ protective oxides compared to CP-Ti and Ti-6Al-4V alloy. Thus, it is expected that the new alloy has improved protective properties in comparison with the reference biomaterials.

### 2.2. Electrochemical Behavior of the Ti-23Hf-3Mo-4Sn Alloy

#### 2.2.1. Electrochemical Behavior from Cyclic Potentiodynamic Curves

**Figure 4:**
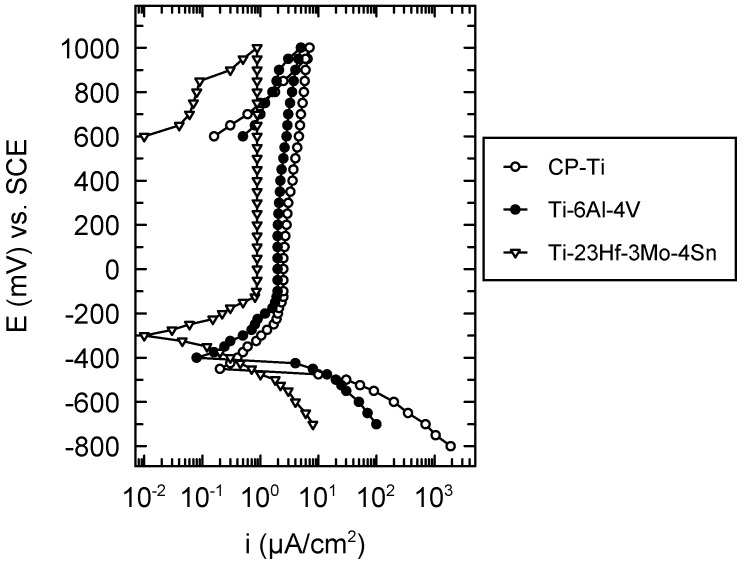
Cyclic potentiodynamic curves for CP-Ti, Ti-6Al-4V and Ti-23Hf-3Mo-4Sn alloys in SBF at 37 °C.

The cyclic potentiodynamic curves presented in Figure 4 evince a typical passive behavior both for CP-Ti, Ti-6Al-4V and the new Ti-23Hf-3Mo-4Sn alloy. These curves did not display hysteresis loops, namely, no local corrosion took place on the surface of the three studied materials; this fact was confirmed by the microscopic observations. As shown in Table 3, more favorable values of all the electrochemical parameters were obtained for the newly developed alloy: more electropositive values of the corrosion, E_corr_, and passivation, E_p_ potentials due to the effect of the galvanic couple of the alloying elements; lower values of the tendency to passivation; |E_corr_ − E_p_| and passive current density; i_p_ which indicates a more rapid, easier, better passivation and a more resistant passive film that prevents the active dissolution of the alloy substrate, respectively [17,18]. The values of all the electrochemical parameters prove a nobler electrochemical behavior of the new alloy than that of CP-Ti and Ti-6Al-4V alloy [19]. This behavior is ascribed to the presence of a thicker native passive film (18.0 nm ± 1.0 nm) on its surface as compared with that of CP-Ti (1.3–3.7 nm) [20] and Ti-6Al-4V (5 nm) [21] alloy; in addition, besides Ti_2_O_3_, TiO_2_ oxides, this film contains HfO_2_, SnO_2_ and MnO_2_ protective oxides that thicken and compact it, conferring very good stability in SBF (see Section 2.1).

#### 2.2.2. Corrosion Resistance from Linear Polarization Tafel Representations

The corrosion parameters i_corr_ (corrosion current density) and V_corr_ (corrosion rate) (Table 4) for the newly developed alloy have lower values of about 8–9 times than those for CP-Ti and Ti-6Al-4V alloys, a fact that demonstrates a more resistant passive film on the new alloy surface. The total quantity of ions released into SBF by the new alloy is lower than that of the reference materials, showing a much reduced toxicity. In addition, polarization resistance, Rp, for the novel alloy has a higher value of about 6–9 times than those of CP-Ti and Ti-6Al-4V alloys due to more protective passive film existing on the new alloy surface [17,18]. The new alloy is placed in the “Perfect Stable” resistance class [22], a fact that depicts a high resistance to corrosion [23]. The higher values of the anodic, β_a_ Tafel slopes than those of the cathodic, β_c_ Tafel slopes reflect the anodic control of the processes from the interface, namely, the existence of the passive layer [24] in addition, to the values of the cathodic β_c_ Tafel slopes around −115 mV/dec. Tafel-like behavior signifies that the cathodic reaction of hydrogen reduction does not depend on the alloy composition [24]. All corrosion parameters confirm a higher protective capacity of the new alloy passive film in comparison with those of the commercial materials CP-Ti and Ti-6Al-4V alloys.

#### 2.2.3. Electrochemical Behavior from EIS

**Figure 5:**
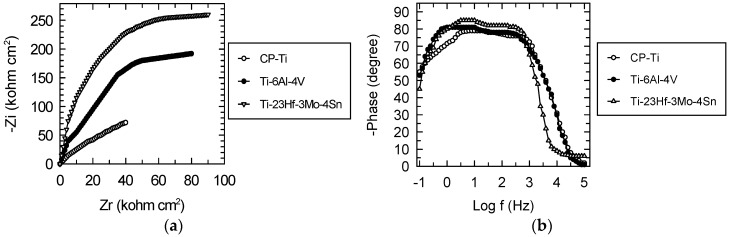
Nyquist and Bode phase angle spectra for CP-Ti, Ti-6Al-4V and Ti-23Hf-3Mo-4Sn alloys in SBF at 37 °C: (**a**) Nyquist plots; and (**b**) Bode phase angle plots.

Nyquist spectra (Figure 5a) are represented by large, incomplete, depressed semicircles, which show a capacitive behavior, and a passive film like an insulator [25,26,27]. The semicircle diameters increase in the order: Ti < Ti-6Al-4V < Ti-23Hf-3Mo-4Sn, namely, the passive film on the new alloy surface possesses the highest insulating, capacitive, protective properties, confirming the XPS results which describe the thickest film.

Bode phase angle spectra (Figure 5b) exhibit two phase angles: in the low frequency range, higher phase angles than those from the middle frequency range can be observed. The values of the first phase angle vary between −79° for Ti, to −81° for Ti-6Al-4V alloy, to −85° for Ti-23Hf-3Mo-4Sn alloy. The highest phase angle for the new alloy denotes the highest capacitive, the most protective passive film [25,26,27,28,29,30,31]; this fact is sustained by the XPS depth profiling analysis that indicates a very thick (18 nm) native passive film. It is known that the thickest passive film assures the highest protection, corrosion resistance [32]. The second, lower angle from the middle frequency range has values of −76° for Ti, −78° for Ti-6Al-4V alloy and −82° for Ti-23Hf-3Mo-4Sn alloy, indicating a defective capacitor with some pores which permit transfer processes from the substrate to solution and from the solution to substrate. Thus, these two phase angles characterize a passive film with two layers [25,26,27,28]: the highest phase angle represents the inner, capacitive, barrier, compact layer and the lower one illustrates the outer, less protective, porous layer that confers bioactivity to the alloy [30,31].

**Figure 6:**
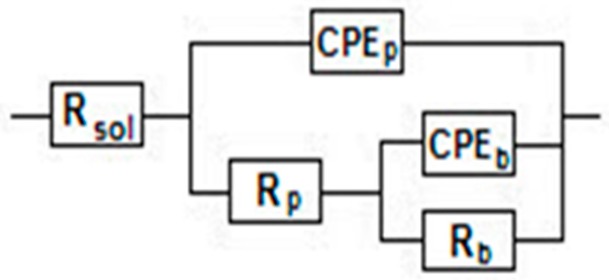
Fitted electric equivalent circuit.

The EIS results were modeled with an electric equivalent circuit consisting of two time constants (Figure 6) as other many authors [25,26,27,28,29,30,31,33,34,35,36]. The electrical parameters are a resistor, R, to show the film conductance and a capacitor, CPE, for the film dielectric properties. The first time constant associated with the high phase angle describes the inner, dense, barrier layer and is composed by the barrier layer resistance, R_b_ and capacitance CPE_b_. The second time constant is related to the lower phase angle that represents the outer, less insulating, less resistant, porous layer and is formed by the porous layer resistance, R_p_ and capacitance, CPE_p_ (the constant phase element, CPE was used instead of capacitance, C to illustrate the non-ideal capacitor).

Fitting parameters from Table 5 have more favorable values for the new Ti-23Hf-3Mo-4Sn alloy, indicating that its passive film is more resistant. This fact confirms the XPS results that revealed a thicker, denser, more compact passive film on the new alloy surface. The inner, barrier layer resistance R_b_ has higher values with two orders of magnitude than the resistance of the outer, porous layer R_p_, denoting that the resistance of the passive film is conferred by the inner, compact layer [25,26]. The barrier layer capacitance, CPE_b_, is one order of magnitude lower than that of the porous layer, CPE_p_, suggesting that the inner layer is thicker than the outer layer [25]. The frequency independent parameter, n, shows the non-uniform current distribution on the surface due to its roughness and homogeneity [25]. For the barrier layer, the n1 values are much closer to 1, namely, an about ideal capacitor; for the porous layer, n2 values are lower, i.e., this layer has a less insulating behavior.

#### 2.2.4. Long-Term Corrosion Resistance from Monitoring of the Open Circuit Potentials

**Figure 7:**
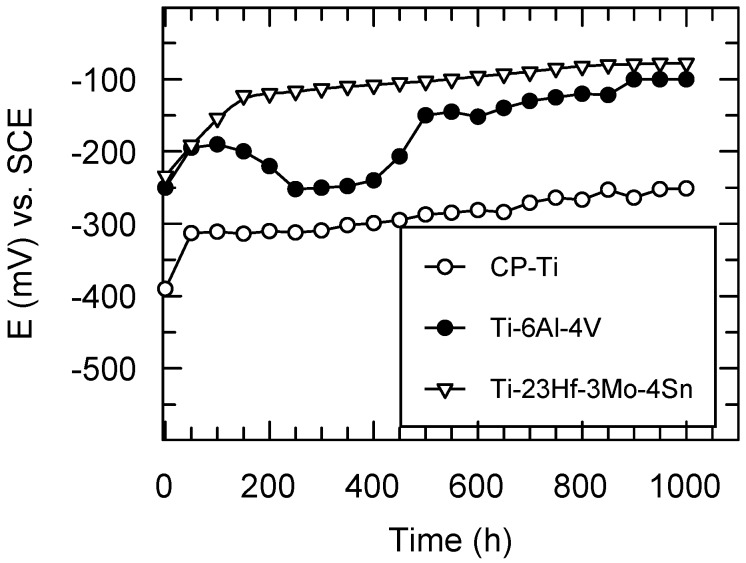
Monitoring of the open circuit potentials for CP-Ti, Ti-6Al-4V and Ti-23Hf-3Mo-4Sn alloy in SBF at 37 °C.

The open circuit potentials (E_oc_) were monitored for 1000 exposure hours of the studied materials exposed to the aggressive action of SBF (Figure 7).

The open circuit potentials for CP-Ti have the most electronegative values from the initial to the final experimental time; the slow increase of E_oc_ values occurred in the first 50 h and then about stable values of −250 mV (vs. SCE) were maintained; these facts point out the slow growth, a low thickening of the passive film followed by a stable passive state [17,18]; the values of E_oc_ for Ti are placed on a Pourbaix diagram [9] in the passive potential range; namely, Ti is passive.

For Ti-6Al-4V alloy, the open circuit potentials have some oscillations at the beginning, reflecting that its passive film is not completely stable [17,18]; after about 500 immersion hours, E_oc_ values slowly move to more electropositive values and then stabilize to about −100 mV (vs. SCE), fact that ascertains a stable passive film [17,18], taking into account that all its constituent elements Ti, Al, V are placed in their passive potential range on Pourbaix diagrams [9].

The novel Ti-23Hf-3Mo-4Sn alloy presents the most electropositive values of its open circuit potentials; these E_oc_ values tend to have nobler values in time, reaching a value of −80 mV (vs. SCE) after 1000 exposure hours in SBF; this behavior reveals a more resistant passive state, namely, the passive film thickened in time and improved its protective properties [17,18]; this fact is normal because all alloying elements Ti, Hf, Sn, Mo find out in their passive state on Pourbaix diagrams [9]. In addition, the new alloy native passive film being thicker than that of the CP-Ti and Ti-6Al-4V alloy confers the best corrosion resistance [25].

From the monitoring of the open circuit potentials, the results showed that the new Ti-23Hf-3Mo-4Sn alloy has the noblest, stable passive behavior.

### 2.3. In Vitro Behavior of Human Umbilical Vein Endothelial Cells

#### 2.3.1. Cell Viability/Proliferation

**Figure 8:**
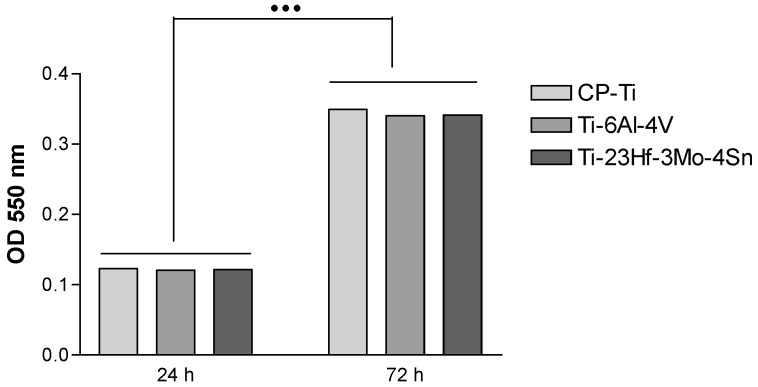
Influence of the test samples on cell proliferation rates as assayed using the MTT colorimetric method. Results demonstrate a time-dependent increase in cell proliferation rate without significant differences between cells growing on the analyzed materials at a certain time point. Data analysis was based on mean ± SD (*n* = 3). ••• *p* ˂ 0.001 between respective groups at 72 h vs. 24 h.

The aim of in vitro research was to assess the biological performance of the new Ti-23Hf-3Mo-4Sn alloy in comparison to commonly used metallic biomaterials CP-Ti and Ti-6Al-4V. Quantification of metabolically active cells was performed for two time points by means of the MTT (3-[4,5-dimethylthiazol-2-yl]-2,5 diphenyl tetrazolium bromide) assay. Results demonstrated an increase in cell proliferation from day 1 to day 3 for all samples (Figure 8). Statistically significant differences among the three groups were not identified. These results demonstrate that the Ti-23Hf-3Mo-4Sn alloy support HUVEC attachment and proliferation.

#### 2.3.2. Expression of the Endothelial Cell Functional Markers

**Figure 9:**
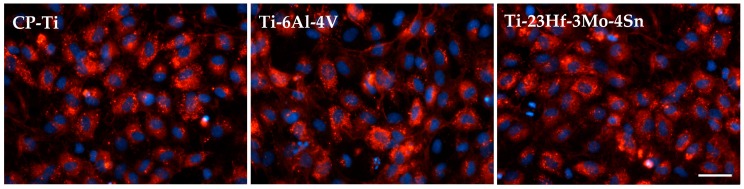
Fluorescent microscopy images of HUVECs cultured for 72 h on CP-Ti, Ti-6Al-4V and Ti-23Hf-3Mo-4Sn surfaces. vWf and cell nuclei were stained **red** and **blue**, respectively. Scale bar represents 50 µm.

Besides cell viability and cell proliferation, considerations of cell phenotype are also important to evaluate cellular response to biomaterials. Therefore, the expression of von Willebrand factor (vWf) and VE-cadherin, specific endothelial cell markers [37,38], was next investigated in order to test whether HUVECs on analyzed materials could maintain a normal endothelial phenotype. The expression and cellular localization of vWf and VE-cadherin were analyzed by immunofluorescence at three days post-seeding. A dotted pattern of vWf was observed within the cytoplasm, mostly in the perinuclear region, in the case of all samples. As shown in Figure 9, cells grown on Ti-23Hf-3Mo-4Sn alloy had similar vWf expression to the cells grown on control samples. Taken together, these results suggest that the newly developed alloy promotes endothelial cell adhesion and proliferation with functional vWf marker expression.

**Figure 10:**
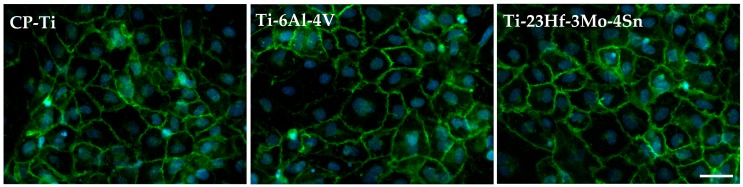
Fluorescent microscopy images of HUVECs cultured for 72 h on CP-Ti, Ti-6Al-4V and Ti-23Hf-3Mo-4Sn surfaces. VE-cadherin and cell nuclei were stained green and blue, respectively. Scale bar represents 50 µm.

Further on, high resolution fluorescence images revealed the expression of VE-cadherin, which was localized to the cell–cell contacts and showed a continuous line of immunofluorescence describing the outer periphery of cells (Figure 10). These results also indicate the preservation of endothelial cells characteristic phenotype on the new alloy.

**Figure 11:**
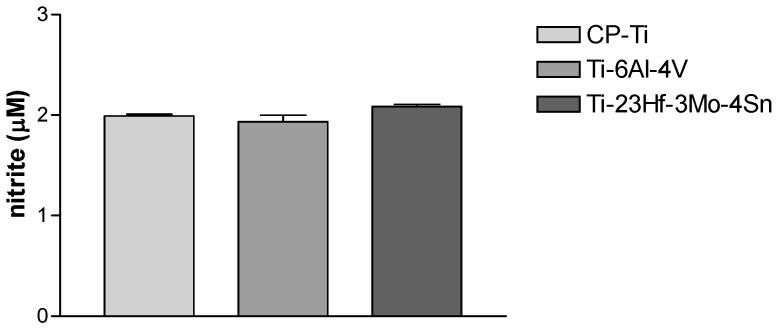
Nitrite concentrations in the cell culture media of HUVECs grown on CP-Ti, Ti-6Al-4V and Ti-23Hf-3Mo-4Sn alloy, as assessed by Griess reagent assay. Data are presented as mean ± SD (*n* = 3).

Another important indicator used to assess the function of endothelial cells adhered to the surface of biomaterials is represented by nitric oxide (NO) production. NO is synthesized continuously by healthy endothelial cells and has a pivotal role in the regulation of vascular tone, vasomotor function, inhibition of leukocyte adhesion to the endothelium, maintaining vascular smooth muscle cells in a non-proliferative state and limiting platelet aggregation [39]. In this study, the level of NO released by the endothelial cells grown on metallic substrates for 72 h was examined by measuring the level of nitrite accumulation in the cell culture media. The results, shown in Figure 11, do not display statistically significant differences between analyzed samples.

## 3. Materials and Methods

### 3.1. Alloy Synthesis

The Ti-23Hf-3Mo-4Sn (at %) ingot was synthesized by cold crucible levitation melting (CCLM) under vacuum, by using a high frequency magnetic induction generator heating system. After melting, a homogenization annealing was performed in the β-phase domain at 1223 K for 72 ks under high vacuum (~10^−7^ mbar) and then quenched in water. After the homogenization step, the ingot was cold rolled up to a reduction level larger than 95% of the initial thickness without intermediate annealing. The final thickness of the cold rolled sheet was about 0.5 mm. Then, disc samples (diameter of about 1.3 cm) were cut from the cold rolled sheet. All samples were then solution treated under high vacuum (~10^−7^ mbar) at 1073 K for 1.8 ks and water quenched at room temperature. Before XPS analysis, electrochemical characterizations and biocompatibility assessment, samples were first mechanically polished on silicon carbide abrasive papers followed by a final mirror polishing step with a colloidal silica suspension (particles size: 50 nm). Roughness of the mirror polished samples was evaluated by atomic force microscopy scans taken in different zones of the surface (AFM, CSM Instrument, tapping mode with a silicon tip).

### 3.2. Alloy Native Passive Film Characterization

XPS technique was used to determine the composition and the thickness of the native passive film existing on the Ti-23Hf-3Mo-4Sn alloy surface. Quantera SXM equipment (Physical Electronics, ULVAC-PHI, Minneapolis, MN, USA) has as an X-ray source, AlKα radiation (1486.6 eV, monochromatized) and an overall energy resolution of 0.75 eV by the full width at half maximum of the Au 4f_7/2_ line. The spectra were calibrated using the C 1s line. The errors in the quantitative analysis (relative concentration) were estimated at ±10% and the accuracy of Binding Energy (BE) assignment was ±2 eV. Before beginning the experiments, a gentle Ar+ ion etching (1 KeV) was applied for 0.2 min aiming to remove the unavoidable surface contaminants and avoid disturbing the surface chemistry.

The oxide thicknesses were calculated by using the sputter rates reported by Baer et al. [14] on a very similar instrument, PHI Quantum 2000 (Physical Electronics, ULVAC-PHI, Minneapolis, MN, USA). To maximize the accuracy of the estimated thicknesses, we used the same setup parameters as in the above-mentioned paper: 2 keV Ar^+^ ion beam, 2 × 2 mm^2^ rastered area, 45° incident angle, and 200 µm spot size diameter. These data were corrected for the escape depth (inelastic mean free path) of the photoelectrons as a function of the kinetic energy.

### 3.3. Electrochemical Characterization

The disc samples were mirror polished and sonochemically degreased in acetone and bi-distilled water for every 30 min, dried in air and then mounted in a tight hold system, and a surface of about 1 cm^2^ was exposed to SBF. The cation and anion concentrations (mmol/L) of SBF in comparison with blood plasma are presented in Table 6. The temperature was kept at 37 °C ± 1 °C.

Three types of the electrochemical polarization tests, cyclic and linear potentiodynamic polarization and EIS were used to determine the passivity and corrosion behavior of the new Ti-23Hf-3Mo-4Sn alloy in comparison with CP-Ti and Ti-6Al-4V alloy. Additionally, the open circuit potentials, E_oc_ were monitored for 1000 immersion hours in SBF (using a performing Hewlett–Packard multimeter=HP34401A, Hewlett-Packard, Palo Alto, CA, USA) to study the materials’ long-term behavior.

The cyclic potentiodynamic polarization was performed from −800 mV (vs. SCE) till +1000 mV (vs. SCE) with a scan rate of 1 mV/s. Voltalab 80 equipment (Radiometer Analytical, Loveland, CO, USA) with its VoltaMaster 4 program (version 7.09, Radiometer Analytical, Loveland, CO, USA) provided the cyclic voltammograms and the following electrochemical parameters: corrosion potential, E_corr_− zero current potential; passivation potential, E_p_− potential for the constant passive current; tendency to passivation, |E_corr_ − E_p_|− low values indicate a very good, easy passivation; passive potential range ΔE_p_− the domain of the constant passive current; passive current density, and i_p_−current density value in the passive potential range. The passive state is characterized by lower values of the current densities on the reverse curve than those on the direct curve; the pitting corrosion is evinced by higher values of the current density on the reverse curve than those on the direct curve [40,41].

The linear polarization was carried-out (with the same Voltalab 80 equipment) for ±150 mV around the open circuit potential using a scan rate of 0.1 mV/s and the VoltaMaster 4 program supplied from Tafel representations of the following corrosion parameters: corrosion current density −i_corr_; corrosion rate −V_corr_; polarization resistance −R_p_; anodic −β_a_ and cathodic −β_c_ Tafel slopes.

EIS was applied with the same Voltalab 80 equipment. Nyquist and Bode spectra were recorded at open circuit potential using a potential signal of 7 mV in a frequency range from 10^−1^ Hz to 10^5^ Hz. The impedance spectra were analyzed by the ZView program (version 3.5a, Scribner Associates Inc., Southern Pines, NC, USA) and an electric equivalent circuit was fitted.

Three samples were used in experiments and the result reproducibility was very good.

### 3.4. Cell Culture

HUVEC cells, purchased from American Type Culture Collection (ATCC), LGC Standards GmbH, Wesel, Germany, were maintained in F-12K Medium (Kaighn’s Modification of Ham’s F-12 Medium) supplemented with 10% fetal bovine serum (Gibco, Grand Island, NY, USA), 1% penicillin−streptomycin (Gibco) and 30 µg/mL endothelial cell growth supplement (Sigma-Aldrich Co., St. Louis, MO, USA) at 37 °C in 5% CO_2_. At approximately 80% confluence, the cells were trypsinized and seeded onto the substrates for cell behavior studies at a density of 10^4^ cells/cm^2^. These studies have been conducted with cells from passage 4 to 6. Prior to cell seeding, samples were sterilized by soaking in 70% ethanol for 30 min. Then, the samples were rinsed twice for 30 min in sterile-filtered MilliQ (Merck Millipore, Darmstadt, Germany) water, air dried and exposed to ultraviolet light in a sterile tissue culture hood, for 30 min on each side. 

### 3.5. Cell Proliferation

Proliferation of HUVECs was monitored at 24 h and 72 h, using MTT assay. Briefly, cell monolayers were incubated with MTT solution (1 mg/ml in serum free culture medium) for 3 h at 37 °C. Then, the MTT solution was decanted and formazan crystals were solubilized with dimethyl sulfoxide. Absorbance of the dye was measured at a wavelength of 550 nm and recorded using a microplate reader (Thermo Scientific Appliskan, Vantaa, Finland).

### 3.6. Immunocytochemical Staining of HUVECs Grown on Ti-23Hf-3Mo-4Sn Alloy

The expression of vWf and VE-cadherin within HUVEC grown on Ti-23Hf-3Mo-4Sn and control surfaces was examined using immunocytochemical staining. Following a 72 h growth period on the samples, the culture media were removed and the cells were washed and fixed by immersion in 4% paraformaldehyde. Following fixation, the cells were permeabilized by incubating with 0.1% Triton X-100 (Sigma-Aldrich Co., St. Louis, MO, USA) in PBS (phosphate-buffered saline), and blocked in PBS containing 2% bovine serum albumin. The samples were then incubated with mouse anti-human vWf monoclonal antibody (Santa Cruz Biotechnology, Dallas, TX, USA) or mouse anti-human VE-cadherin monoclonal antibody (Santa Cruz Biotechnology) in PBS containing 1.2% BSA (bovine serum albumin). After washing with PBS, they were further incubated with Alexa Fluor 546-conjugated goat anti-mouse IgG antibody ( Molecular Probes, Eugene, OR, USA) and Alexa Fluor 488-conjugated goat anti-mouse IgG antibody ( Molecular Probes), respectively, in PBS containing 1.2% BSA, followed by washing with PBS. A 2 µg/mL DAPI (4′6-diamidino-2- phenylindole) solution was used to stain cell nuclei. Fluorescent images were taken with an Olympus IX71 inverted microscope (Olympus, Tokyo, Japan).

### 3.7. NO Release Assay

To detect the release of NO, HUVECs were seeded onto substrates placed in 24 well plates at a density of 5 × 10^4^ cells/cm^2^. After 3 days of culture, the supernatants of each well were collected. The relative content of NO in the supernatants was measured as previously described [42].

### 3.8. Statistical Analysis

For the statistical analysis of the MTT and NO release assays, one-way ANOVA with Bonferroni’s multiple comparison tests (GraphPad Prism software, Version 3.03, GraphPad, San Diego, CA, USA) was performed. A probability of *p* < 0.05 was considered significant.

## 4. Conclusions

XPS survey spectra acquired on the novel Ti-23Hf-3Mo-4Sn alloy surface showed that the thickness of the native passive film is of ~18.0 nm.

The deconvoluted spectra evinced that this surface film consisted of protective TiO_2_, Ti_2_O_3_, HfO_2_; MoO_2_, and SnO_2_ oxides. The thicknesses of the individual oxides are quite different, HfO_2_ being the main contributor to the overall thickness of the film on the alloy surface.

The values of all the electrochemical parameters prove a nobler electrochemical behavior of the new superelastic Ti-23Hf-3Mo-4Sn alloy by comparison with CP-Ti and Ti-6Al-4V alloy. This behavior is ascribed to the favorable influence of the alloying elements Hf, Sn, and Mo which enhanced the protective properties of the alloy native passive film. All corrosion parameters for the new alloy confirm a higher protective capacity that prevents the dissolution of the alloy substrate in comparison with commercial materials CP-Ti and Ti-6Al-4V alloy.

Nyquist and Bode impedance spectra characterized a passive film with two layers, namely an inner, capacitive, barrier, compact layer and an outer, less protective, porous layer; these two layers confer both protection and bioactivity to the alloy.

Monitoring of the open circuit potentials resulted in the new Ti-23Hf-3Mo-4Sn alloy having the noblest, stable behavior, and more resistant passive film than those in the reference materials.

Ti-23Hf-3Mo-4Sn surface displayed excellent endothelial cell growth. Moreover, HUVEC cells retained their phenotype and functions as revealed by vWf, VE-cadherin expressions and NO assay.

Overall, the results obtained in the present study demonstrate that this new superelastic Ti-23Hf-3Mo-4Sn alloy represents a promising material for various medical devices, particularly for the manufacture of self-expanding vascular stents or superelastic catheters for which a perfect biocompatibility and a large recovery strain are required.

## Figures and Tables

**Table 1:** XPS data of Ti-23Hf-3Mo-4Sn alloy surface chemistry: the binding energies vs. ions.

**Ions**				
O 1s	Ti 2p_3/2_	Hf 4f_7/2_	Mo 3d_5/2_	Sn 3d_5/2_
**Binding energies (eV)/Ions**				
530.5/O^2−^	457.0/Ti^3+^	17.0/Hf^4^	228.1/Mo^0^	484.7/Sn^0^
531.9/OH ads	458.9/Ti^4+^		228.8/Mo^4+^	486.7/Sn^4+^

**Table 2:** XPS data of Ti-23Hf-3Mo-4Sn alloy surface chemistry: relative concentration (%) vs. oxidation states.

**Ions**			
Ti	Hf	Mo	Sn
**Concentration (%)/Oxidation States**			
21.3/Ti^3+^	100.0/Hf^4+^	8.4/Mo^0^	13.0/Sn^0^
78.7/Ti^4+^		91.6/Mo^4+^	87.0/Sn^4+^

**Table 3:** Electrochemical parameters for CP-Ti, Ti-6Al-4V and Ti-23Hf-3Mo-4Sn alloys obtained from cyclic potentiodynamic polarization tests in SBF at 37 °C.

Material	E_corr_ (mV)	E_p_ (mV)	ΔE_p_ (mV)	|E_corr_ − E_p_| (mV)	i_p_ (µA/cm^2^)
CP-Ti	−470	−150	>1000	320	2.512
Ti-6Al-4V	−400	−150	>1000	250	1.995
Ti-23Hf-3Mo-4Sn	−300	−100	>1000	200	0.873

**Table 4:** Corrosion parameters for CP-Ti, Ti-6Al-4V and Ti-23Hf-3Mo-4Sn alloys obtained from potentiodynamic linear polarization tests in SBF at 37 °C.

Material	i_corr_ (µA/cm^2^)	V_corr_ (µm/Y)	Ion Release (ng/cm^2^)	Resistance Class	R_p_ (kΩ·cm^2^)	β_a_ (mV/dec)	β_c_ (mV/dec)
CP-Ti	0.552	5.051	513.18	VS	20.52	180	−119
Ti-6Al-4V	0.492	4.502	457.40	VS	30.75	189	−118
Ti-23Hf-3Mo-4Sn	0.061	0.649	65.93	PS	198.18	191	−115

VS—Very Stable; PS—Perfect Stable.

**Table 5:** Fitting parameters of the electric equivalent circuit for CP-Ti, Ti-6Al-4V and Ti-23Hf-3Mo-4Sn alloys in SBF at 37 °C.

Material	R_s_ (Ω·cm^2^)	R_b_ (Ω·cm^2^)	CPE_b_ (S·s^n^cm^−2^)	n1	R_p_ (Ω·cm^2^)	CPE_p_ (S·s^n^cm^−2^)	n2
CP-Ti	12.4	8.3 × 10^5^	9.4 (±0.1) × 10^−6^	0.95	7.2 × 10^3^	1.8 (±0.1) × 10^−5^	0.88
Ti-6Al-4V	13.6	9.5 × 10^5^	9.2 (±0.2) × 10^−6^	0.97	1.8 × 10^4^	1.1 (±0.1) × 10^−5^	0.90
Ti-23Hf-3Mo-4Sn	14.5	1.7 × 10^6^	8.1 (±0.1) × 10^−6^	0.99	1.9 × 10^4^	0.9 (±0.1) × 10^−5^	0.91

**Table 6:** Ion concentrations (mmol/L) of SBF and human blood plasma.

Ion	Simulated Body Fluid	Blood Plasma
Na^+^	142.0	142.0
K^+^	5.0	5.0
Mg^2+^	1.5	1.5
Ca^2+^	2.5	2.5
Cl^−^	148.8	103.0
HCO_3_^−^	4.2	27.0
HPO_4_^2−^	1.0	1.0
SO_4_^2−^	0.5	0.5
